# Inflammatory endobronchial polyps unleashing recurrent pneumothorax: A case report

**DOI:** 10.1002/rcr2.1278

**Published:** 2024-01-17

**Authors:** Ummi Nadira Daut, Muhammad Halmii Faisal Thena, Tan Hui‐Xin, Mona Zaria Nasaruddin, Jamalul Azizi Abdul Rahaman

**Affiliations:** ^1^ Medical Department University Putra Malaysia Selangor Malaysia; ^2^ Pulmonology Department Hospital Sultan Idris Shah Serdang Serdang Malaysia

**Keywords:** debulking, endobronchial inflammatory polyp, pneumothorax, rigid bronchoscopy

## Abstract

Inflammatory endobronchial polyps (IEPs) are rare benign lesions that originate from the bronchial mucosa. While pneumothorax is a well‐known complication of various pulmonary conditions, its association with IEPs is exceedingly uncommon and poorly understood. This case report presents a unique and explosive encounter of a patient with an inflammatory endobronchial polyp who experienced a pneumothorax, shedding light on the clinical presentation, diagnostic challenges, and management strategies for this rare entity.

## Graphical abstract

This case report explores the rare association between inflammatory endobronchial polyps (IEPs) and recurrent pneumothorax. The report presents the case of a 30‐year‐old woman who was diagnosed with IEPs obstructing the bronchi, leading to recurrent pneumothorax. The report highlights the diagnostic challenges, management strategies, and the need for further research to understand this uncommon association.

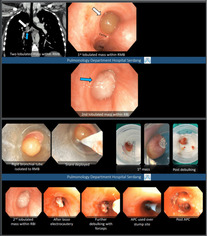

## INTRODUCTION

Inflammatory endobronchial polyps are non‐neoplastic, inflammatory lesions that arise from the bronchial mucosa. They present with symptoms such as cough, dyspnea, and recurrent respiratory tract infections. However, pneumothorax as a presenting feature of IEPs is an exceptionally rare occurrence, with only a few cases reported in the medical literature. This case report aims to expand our understanding of this unusual association and explore the diagnostic and therapeutic considerations.

## CASE REPORT

**FIGURE 1:**
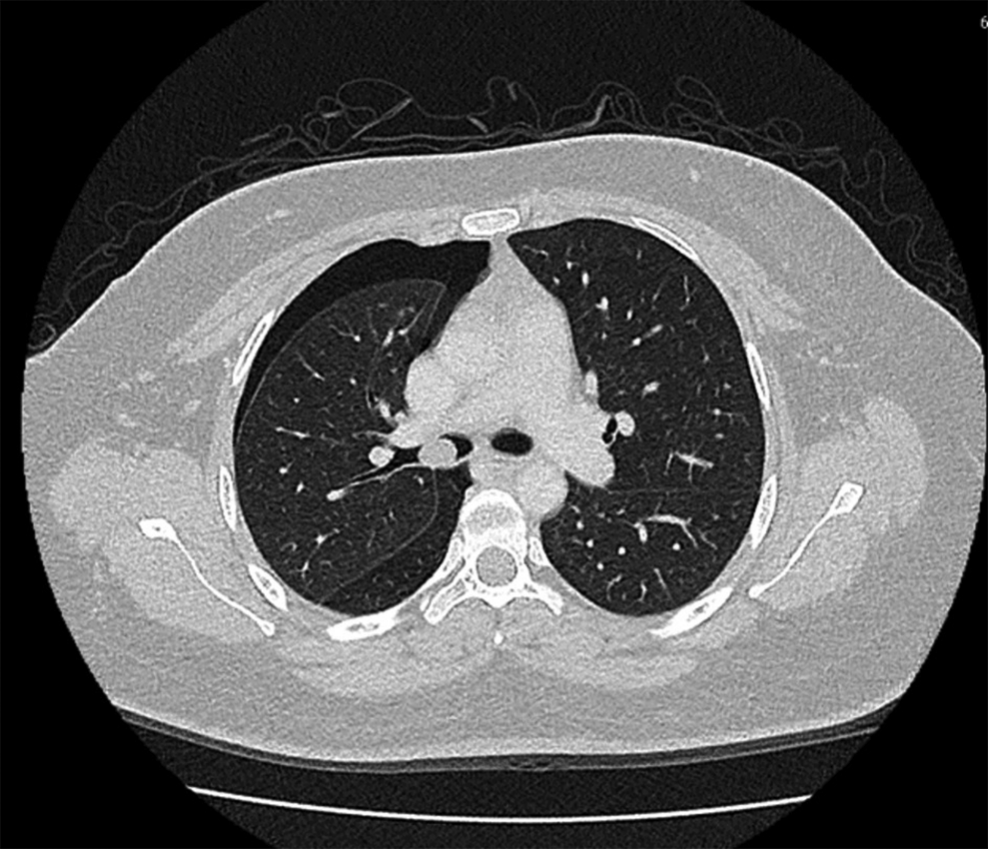
Computed tomography thorax with right endobronchial lesion and right sided pneumothorax.

**FIGURE 2:**
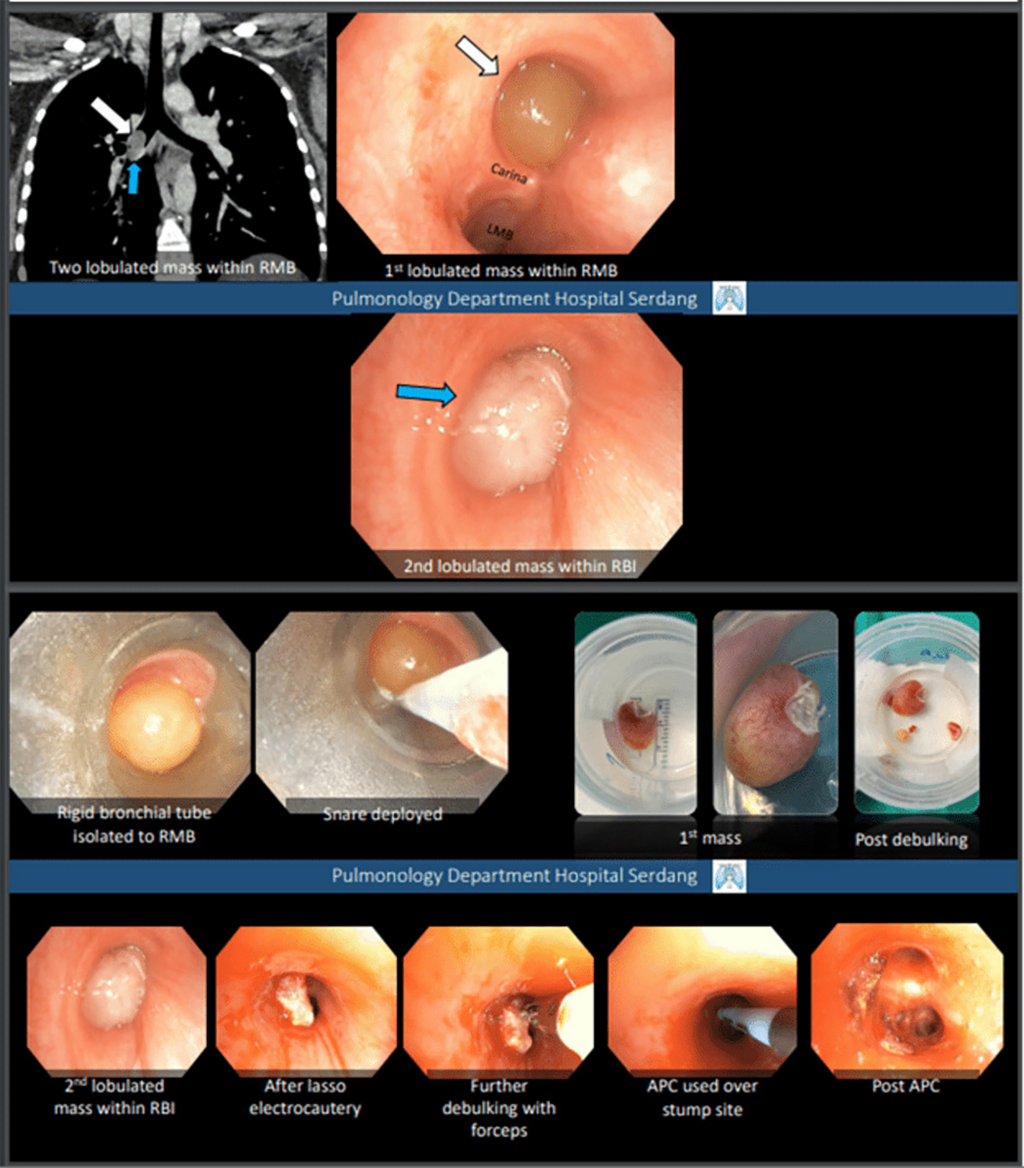
Bronchoscopy finding revealed two lobulated mass within the right main bronchus and bronchus intermedius. Rigid bronchial tube was inserted at right mainstem bronchus. Snare was deployed followed by lasso electrocautery and debulking of the mass with forceps. Argon plasma coagulation (APC) was used over the stump site and post APC showed minimal residual lesions.

We present a case of a 30‐year‐old woman with no significant medical history who presented with sudden onset right‐sided chest pain, shortness of breath, and wheezing. Upon examination, reduced breath sounds, and generalized rhonchi were observed on the right side. Chest radiography revealed a right‐sided pneumothorax, which was managed with chest tube insertion. The patient experienced a second episode of pneumothorax after 17 days, prompting further investigation. Computed tomography (CT) thorax demonstrated a non‐enhancing, non‐radiopaque filling defect within the right main bronchus, obstructing the upper and descending bronchi with recurrent pneumothorax (Figure 1). Bronchoscopy revealed polypoidal mass obstructing right mainstem bronchus. The patient subsequently underwent rigid bronchoscopy. Two lobulated polypoidal masses were removed using electrocautery snare followed by debulking using forceps and using argon plasma coagulation (APC) over the stump site (Figure 2). Histopathological analysis confirmed the presence of endobronchial inflammatory polyps. Surveillance bronchoscopy revealed no recurrence.

## DISCUSSION

Endobronchial polyps found in the tracheobronchial tree are uncommon nonneoplastic lesions with distinct histopathological characteristics. They can be categorized into multiple papillomas, solitary papillomas, and inflammatory polyps. From a previous study, the most common benign neoplasm was hamartoma (37%), followed by lipoma (19%), squamous papilloma (11%), pleomorphic adenoma (7%), mucin gland adenoma (7%), papillary adenoma (3%), hemangioma (3%), neurofibroma (3%), leiomyoma (3%), and papillomatosis (3%) (1).

The association between IEPs and pneumothorax is a rare phenomenon, with limited literature available. There was a similar case of a patient with underlying emphysema that presented with spontaneous pneumothorax and was found to have left upper lobe endobronchial lesion causing obstruction of the bronchus. The biopsy was consistent with diagnosis of fibrous stromal polyp (2).

The exact pathophysiology underlying this relationship remains unclear. Proposed mechanisms include air trapping distal to the obstructing polyp, leading to increased pressure and subsequent rupture of alveoli. From previous literature, foreign body in the airway behaves as a check valve (3, 4) causing a similar mechanism. Additionally, mechanical disruption of the mucosal membrane of the airway (3) leads to pneumomediastinum and pneumothorax. In inflammatory endobronchial polyps, the inflammatory changes in the bronchial wall may weaken the integrity of the lung parenchyma, which also predisposes to pneumothorax. The recurrence rate of pneumothorax post resection of the endobronchial lesion is unknown. The recurrence depends on various factors, including the underlying cause, the type of procedure performed, and individual patient characteristics.

In most of the case reports, cough, dyspnea, fever, pseudo‐asthmatic wheeze, or rarely hemoptysis are common presenting symptoms. A previous case reports of an elderly male presenting with prolonged cough and a recurrent lower respiratory tract infection. On evaluation, the patient was found to have an inflammatory polyp that was removed completely using electrocautery snare through a flexible bronchoscope (5).

Diagnosing IEPs can be challenging, as they can mimic other bronchial pathologies. The differential diagnosis for endobronchial mass includes bronchial carcinoid tumour, lung malignancy, hamartomas, infectious causes such as fungal infections, Tuberculosis, inflammatory conditions such as Granulomatosis with polyangiitis (Wegener's), Sarcoidosis and inhaled foreign body. Hemangiomas is also one of the possible diagnoses. Flexible bronchoscopy is the gold standard for visualizing and obtaining biopsy specimens. Prompt recognition of pneumothorax and appropriate management, such as thoracostomy tube insertion, is crucial to relieve symptoms and prevent complications. Surgical resection of the polyp may be considered in cases of recurrent pneumothorax or persistent symptoms.

Electrocautery snare has been used widely for endobronchial resection and help to obtain diagnosis and achieve complete resection in a single procedure. It serves as a potential alternative to surgery and moving forward. Previous case report successfully showed resection of IEP with electrocautery snare on index bronchoscopy (6).

The prognosis for IMT can vary widely based on several factors, including the location of the tumour, its size, and whether it is benign or malignant. In many cases, IMT is a benign condition (7), and complete surgical resection is often curative. Benign IMTs tend to have a favourable prognosis, with a low likelihood of recurrence after successful surgical removal. In some instances, IMT can exhibit more aggressive behaviour, with local invasion or recurrence. Malignant forms of IMT may require more aggressive treatments, such as additional surgery, radiation therapy, or chemotherapy. The prognosis for malignant IMT can be less favourable than for the benign form. In smaller and non‐urgent polyps, inhaled corticosteroids are the therapeutic option in inflammatory bronchial polyps, especially in cases where the patient has asthma as an underlying condition (8).

In conclusion, this case highlights the importance of considering inflammatory endobronchial polyps as a potential aetiology in patients presenting with pneumothorax. It underscores the need for further research to elucidate the underlying mechanisms and optimize diagnostic and management approaches. Increasing awareness of this rare association can aid clinicians in making accurate diagnoses and providing timely interventions for improved patient outcomes.

## AUTHOR CONTRIBUTIONS

Ummi Nadira Daut: Conceptualization, methodology, and writing‐original draft preparation. Muhammad Halmii Faisal Thena, Tan Hui‐Xin, Mona Zaria Nasaruddin, Jamalul Azizi Abdul Rahaman: Review and supervision. All authors have read and agreed to the published version of the manuscript.

## FUNDING INFORMATION

The financial support for the publication of this manuscript has been equally shared among all authors.

## CONFLICT OF INTEREST STATEMENT

None declared.

## ETHICS STATEMENT

The authors declare that appropriate written informed consent was obtained for the publication of this manuscript and accompanying images.

## Data Availability

Data is not shared due to confidentiality and privacy restrictions.

## References

[1] Insler JE , Seder CW , Furlan K , Mir F , Reddy VB , Gattuso P . Benign endobronchial tumors: a clinicopathologic review. Front Surg. 2021;8. 10.3389/fsurg.2021.644656 PMC7973360 33748183

[2] Akkanti B , Santacruz F , Herlihy P . Endobronchial stromal polyp causing pneumothora. Chest. 2011;140(4):140A.

[3] Velecharla MS , Shah KD , Bradoo RA , Subramaniasami GS , Joshi AA . “AIR LEAK SYNDROME”: an unusual presentation of foreign body in the airway. Indian J Otolaryngol Head Neck Surg. 2019;1(71):693–695. 10.1007/s12070-018-1495-0 PMC6848634 31742044

[4] Matsuura H , Inoue S , Atagi K , Kawaguchi M . Life‐threatening check valve formation due to tracheobronchial aspergillosis. JA Clin Rep. 2015;1(1):17. 29497649 10.1186/s40981-015-0022-5 PMC5818705

[5] Kuwal A , Advani M , Mathur DR . Benign endobronchial inflammatory polyp with cystic degeneration: a case report. Turk Thorac J. 2019;20(4):258–261. 31584388 10.5152/TurkThoracJ.2019.180173 PMC6777656

[6] Tosonian S , Callison JC . Endobronchial fibroepithelial polyp diagnosed and treated with electrocautery snare [Internet]. www.atsjournals.org

[7] Casanova M , Brennan B , Alaggio R , Kelsey A , Orbach D , van Noesel MM , et al. Inflammatory myofibroblastic tumor: the experience of the European pediatric soft tissue sarcoma study group (EpSSG). Eur J Cancer. 2020;127:123–129. 32007712 10.1016/j.ejca.2019.12.021

[8] Niimi A , Amitani R , Ikeda T , Kubo Y , Tanaka E , Kuze F . Inflammatory bronchial polyps associated with asthma: resolution with inhaled corticosteroid. Eur Respir J. 1995;8(7):1237–1239. 7589412 10.1183/09031936.95.08071237

